# Non-Linear Template-Based Approach for the Study of Locomotion

**DOI:** 10.3390/s20071939

**Published:** 2020-03-30

**Authors:** Tristan Dot, Flavien Quijoux, Laurent Oudre, Aliénor Vienne-Jumeau, Albane Moreau, Pierre-Paul Vidal, Damien Ricard

**Affiliations:** 1 Université Paris-Saclay, ENS Paris-Saclay, CNRS, Centre Borelli, F-94235 Cachan, France; 2 Université de Paris, CNRS, Centre Borelli, F-75005 Paris, France; 3 ORPEA Group, F-92813 Puteaux, France; 4 Université Sorbonne Paris Nord, L2TI, UR 3043, F-93430 Villetaneuse, France; 5 Service de Neurologie, Service de Santé des Armées, Hôpital d’Instruction des Armées Percy, F-92190 Clamart, France; 6 Hangzhou Dianzi University, Hangzhou C-310005, China; 7 Ecole du Val-de-Grâce, Ecole de Santé des Armées, F-75005 Paris, France

**Keywords:** step detection, pattern recognition, gait analysis, Dynamic Time Warping

## Abstract

The automatic detection of gait events (i.e., Initial Contact (IC) and Final Contact (FC)) is crucial for the characterisation of gait from Inertial Measurements Units. In this article, we present a method for detecting steps (i.e., IC and FC) from signals of gait sequences of individuals recorded with a gyrometer. The proposed approach combines the use of a dictionary of templates and a Dynamic Time Warping (DTW) measure of fit to retrieve these templates into input signals. Several strategies for choosing and learning the adequate templates from annotated data are also described. The method is tested on thirteen healthy subjects and compared to gold standard. Depending of the template choice, the proposed algorithm achieves average errors from 0.01 to 0.03 s for the detection of IC, FC and step duration. Results demonstrate that the use of DTW allows achieving these performances with only one single template. DTW is a convenient tool to perform pattern recognition on gait gyrometer signals. This study paves the way for new step detection methods: it shows that using one single template associated with non-linear deformations may be sufficient to model the gait of healthy subjects.

## 1. Introduction

Human locomotion is a complex mechanism, the analysis of which is essential. Indeed, age and many pathologies (such as Parkinson’s disease, arthritis, multiple sclerosis, stroke, obesity, diabetes, etc.) can alter human gait, resulting in increased fall-risk and decreased autonomy. Precise quantification of the different gait phases would therefore improve doctors’ diagnostics, understanding of pathologies and risk prevention. Healthy gait is composed of a repetition of similar patterns. Each step is composed of a succession of key instants. Those instants are, in their order of occurrence: Heel Strike (Initial Contact, IC), Foot Flat, Heel Off and Toe Off (Final Contact, FC). The detection of steps, and their key instants, is a basic building block necessary to calculate clinical features of interest.

To study those steps and their associated gait events, different sensors and algorithms can be used. Among these solutions can be found instrumented mats, force platforms, camera-optical tracking systems and force-sensitive resistors insoles. They have been used successfully in several studies and are often considered as the gold standard for gait events detection [1,2,3,4]. In the last past ten years, Inertial Measurement Units (IMU) have gained popularity in this domain. These low-cost sensors, composed of accelerometers, gyroscopes and magnetometers, have been used in clinical studies [1,5,6,7,8,9,10] with good results. Those IMU present multiple advantages: they are low-cost, small in size (easy to use in clinical settings), and are body-worn (they do not require any specific installation). They can be attached at the foot, shank, thigh, wrist or chest of the studied subjects.

The majority of step detection techniques from IMU data rely on filtering, thresholding, zero-crossing or peak detection techniques that are applied on the accelerometer/gyrometer signals [1,6,11,12]. After a preprocessing stage, a specific event, supposedly characteristic of the step, is detected. The most well-known preprocessing stage, initially designed for ECG signals, was introduced by Pan-Tompkins [13]. It has been used in multiple step-detection methods [6,12,14,15]. Unfortunately, all these methods depend on the tuning of several parameters (width of the bandpass filter, thresholds, etc.), which are often set according to empirical experience, and heavily depend on the type of cohort [6]. For these reasons, the use of templates has been advocated in several recent articles [12,16,17,18]. Template-based step detection has also been used to compare gait trials [19]: the concept of template appears as a convenient tool for the study of locomotion and steps.

The use of templates for the study of locomotion is actually not novel. Murray in the 1960s [20] already put forward the idea of a walking pattern in the healthy subject, which was used to specify ranges of values for each joint involved in walking [21]. Although normative values can be found, this pattern is also specific to each individual. This idea has been used extensively since then for gait recognition, allowing individuals to be recognised by the way they walk [22]. The analysis of the individual gait pattern has gained in popularity with the identification possibilities it allows, particularly through video cameras [23,24,25,26]. However, the task remains tricky and an open problem since there exists a distinction between the individual gait pattern and the general characteristics shared by a group during walking. In addition, it has been shown that determining the individuality of a walking pattern from a single sample per person is not easy [27].

The use of machine learning algorithms has shown its usefulness in the search for an individual gait pattern and its evolution over different time scales [28]. This gait pattern recognition can be used to successfully solve tasks for classifying gait disorders [29,30,31,32] or for extracting gait characteristics [33]. Deep learning methods have also been used to characterise the gait phase the subject is in, thus resulting in IC/FC detection from multiple accelerometers [34], 3D markers [35,36] or instrumented shoes [37].

This article is aimed at studying the notion of step template in healthy gait signals. We investigate whether it is possible to accurately detect all the steps, of different subjects, thanks to a single step template of gyrometer signal. This hypothesis is not as innocuous as it might seem. Indeed, in previous methods, a database of several templates, representative of several steps from patients with known pathologies, was used by the template-matching algorithm [16]. Successfully reducing this multiple-templates database back to a single template would not only have positive computational consequences, but also be very interesting in a theoretical point of view, for a better neurological understanding of human gait. Indeed, if a single step template is enough to accurately detect all the steps of different healthy subjects, it implies that these subjects share a common locomotion pattern, as first intuited by Murray [20].

Provided one such pattern does exist, what kind of deformations does it undergo among the healthy population? Particularly, some measures of fit (the Euclidian distance between signals, the Pearson correlation, etc.) must be used to compare the time series shapes. Those measures are based on sample-by-sample distances: as such, they require a common time scale between studied signals. This condition is rather unlikely when considering different subjects who walk at different speeds and with different gait style. In particular, in this article, we investigate a warping of the time series based on the popular Dynamic Time Warping (DTW) algorithm, which assumes non-linear time deformations.

The contributions of this article are:

- A novel template-based step detection algorithm based on DTW, that assumes non-linear time deformations between templates
- The introduction of several strategies for inferring and learning a step pattern from gyrometer data
- A discussion on the role and the use of templates for the study of locomotion

This article is articulated as follows. Section 2 presents some background knowledge on gait events and bio-mechanical structure of steps as well some mathematical insights on DTW. Section 3 details the protocol, data and population used in this study. Section 4 describes the methodology for DTW-based step detection and pattern inference from gyrometer data. Several strategies using both linear and non-linear realignment are presented. These strategies and proposed algorithm are compared and tested in Section 5 and Section 6.

## 2. Background

This section aims at summarising several important notions for the understanding of this paper. Section 2.1 gives an overview of the biomechanical phenomena occurring during healthy locomotion and Section 2.2 reviews the principle of Dynamic Time Warping (DTW).

### 2.1. Gait Events

**Figure 1:**
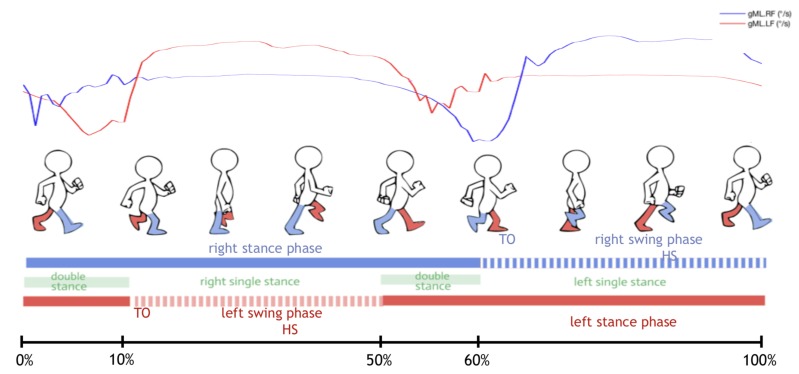
Key phases of the Gait Cycle, represented schematically, and put in parallel of angular velocity signals, obtained by two foot-mounted Xsens sensors.

A single gait cycle is defined as the period between two successive repetitive gait events [38]. It consists of two main phases, namely the support and oscillation phases. The support phase begins with initial contact (IC), which marks the beginning of the transfer of the load to the foot of contact with the ground. The support phase ends with the foot-off (FO), which marks the complete elevation of the foot. The oscillation phase begins with the FO and ends with the IC, which is the phase of swinging the foot parallel to a forward movement of the body. Normally, the support phase constitutes about 60% of the entire walking cycle, and the oscillation phase the remaining 40% [38]. From now on, we only focus on the support phase in order to focus on the detection of steps. We divide the position-taking phase into five events [39]: Heel Strike (HS), Foot Flat (FF), Midstance (MS), Heel Off (HO) and Toe Off (TO). These phases are illustrated in Figure 1 for foot-mounted gyrometers.

#### 2.1.1. HS to FF

The first phase begins with the attack of the heel and the lowering of the foot to the ground (between 0% and 12% of the cycle) [40]. At HS, we can see a short velocity peak of the opposite sign just before its drop to a larger negative peak. From data presented previously by Cloete and Scheffer (2010) [41], the observed peak of angular velocity following HS is present just before the deceleration of the tibio-tarsal joint. This phase corresponds to the loading phase; its role is to transfer the weight from the body to the supporting leg, to maintain the speed and balance of the body’s centre of gravity by absorbing energy through a braking action of the leg muscles [42]. Indeed, the foot continues towards the ground immediately after the HS, the dorsiflexors controlling this plantar flexion movement to prevent the foot from dropping while allowing it to roll to the position of the flat foot. Otherwise, if the dorsiflexors are unable to slow down the movement, we speak of a foot drop [43]. The braking is represented by the reduction of the angular velocity after the negative peak. The contact of the heel with the ground is lateral to the centre of the ankle joint. Thus, the weight of the body is transmitted to the astragalus, creating a pronatory moment in the subtalar joint which, in turn, stresses the structures of the medial arch. The HS can be detected by the characteristic lateral angular velocity peaks with inertial units mounted on the instep [44].

#### 2.1.2. FF to MS

From 12% to 34% of the cycle occurs the intermediate support phase which corresponds to the response of the ground on the foot and the first half of the unipodal support. During this phase, there is a transfer of the load from the body to the supporting leg, allowing the body to move forward above the foot. This phase ends when the body’s centre of gravity is vertical to the foot. Its role is to anchor the foot to the ground so that it becomes the pivot of the leg supporting the body’s weight. During this phase, the stability of the foot makes it possible to control the correct movement of the leg around this axis. We observe a return to a plateau value close to zero for the angular velocity of the foot after the FF, in the sagittal plane.

#### 2.1.3. MS to HO

The end-of-support phase is carried out from 34% to 50% of the cycle and corresponds to the second half of the unipodal support. During this phase, the foot is flat, the angles around the anteroposterior and mediolateral axes must be constant, but the gyroscopes show that there are slight disturbances. This is mainly because the body’s centre of gravity moves forward during the flatfoot phase, causing the shoes to deform, so that the orientation of the sensor changes [45].

#### 2.1.4. HO to TO

The second bipodal support is performed from 50% to 62% of the cycle and is called the pre-oscillating phase for the foot that was in support until then [40]. The body weight is then transferred to the other leg which starts its support phase. Its role is to push the leg forward by propelling the forefoot against the ground. After the heel is raised, the ankle joint returns to plantar flexion, forcing the metatarsophalangeal joints to dorsiflexion [43]. At HO, the angular velocity drops to negative values. At the time of HS, the value of the angular velocity of the foot drops to negative values related to the detachment of the heel from the ground and the change of axis of rotation around the ends of the metatarsals. A gyroscope attached to the instep shows a high speed in connection with the extension of the tibiotarsal joint during this propulsion phase. After the TO, the leg turns inward, pronouncing the foot again and unlocking the transverse tarsal joint so that the foot returns to its flexible state for the oscillating phase. The vertical movement of the heel begins well before the TO and reaches its maximum upward speed just before the toes come off. Based on a minimum speed threshold at the support phase, Sabatini et al. (2005) [46] sought the transition from HO to TO for their step detection algorithm. This transition occurs when the angular velocity reaches the maximum negative value. For Jasiewicz et al. (2006) [47], this negative angular value signs the TO and thus the beginning of the oscillating phase which will end with a return to zero on the new initial contact (HS).

To summary, plantar flexion starts hence at the heel strike, which can be associated with a peak acceleration due to both the impact against the ground and the change of pivot during the heel support. Immediately after HS, deceleration due to dorsiflexors activity (especially the anterior tibialis) will reverse the angular velocity curve. This speed decreases during deceleration of the forefoot when it is lowered against the ground. The pivot phase aforementioned between 12% and 34% of the walking cycle, when the foot is used as a support for the loading of the hemibody, leads to a plateau phase of the angular rotation of the forefoot. The second half of the support phase can be characterised by slight variations recorded by gyroscopes. In the pre-oscillating phase, the angular velocity becomes negative again with the start of plantar flexion, which accelerates the movement to propel the body forward and advancing the centre of pressure to the toes. As the foot is released from the ground thanks to a new plantar flexion, there is an inversion of the angular velocity on the end toe-off.

### 2.2. Dynamic Time Warping

Dynamic Time Warping (DTW) algorithm is used to find time correspondences between time series in an automatic manner and with a very good precision. It can be used to align in time two temporal sequences, or to evaluate how close two time series are to one another, independently of time fluctuations. Historically, it was introduced in the early 1980s for word recognition, where the speed and pronunciation might essentially differ between speakers [48,49]. Then, it appeared to be useful in a large range of domains, from finance [50] to electrocardiograms (ECG) signals changes [51].

The DTW distance measure, combined with a nearest neighbor classifier, is considered as one of the best current choices for time series classification, giving, at least, state-of-the-art performances [52,53,54,55,56]. Even if some distances (mostly derived from DTW), as the ShapeDTW measure [57], are able to slightly outperform DTW, it is still one of the strongest measures for time series classification. DTW algorithm has already been used to characterise electrocardiograms (ECG) signals, and particularly as a tool in Pattern Recognition of ECG Changes [51,58]. Interestingly, the DTW algorithm is able to outperform wavelet analysis in the classification and identification of ECG of some heart disturbances [58]. DTW has also been historically used for gene-expression time-analysis [59], and, more recently, in the study of temporal patterns in patient diseases trajectories [60] or pattern retrieval [54,61]. Recently, DTW algorithm has also been used to create an adapted library of signals for step detection [62] and for stride segmentation during daily life activities [63,64].

#### 2.2.1. Principle and Algorithm

DTW algorithm is based on the search of an optimal matching path between two time series. The algorithm searches for all the different paths possible between the two times series, i.e., all the different pairs of element-to-element matches possible between the two time series. Then, the path with the minimum global (i.e., cumulative) distance is selected, and constitutes the “warping path” between the two times series. On this path, equivalent features of the two time series appear at the same location, highlighting the similarities between the signals. The cumulative distance between the pairs of the optimal path corresponds to the DTW distance measure (it is a distance-like quantity, but it does not guarantee the triangle inequality to hold). We can note here that the complexity of this algorithm is quadratic (in relation to the number of samples of the longest time series).

More specifically, considering two multivariate time series u and v in Ω and of respective lengths $N_{u}$ and $N_{v}$, the aim of the algorithm is to build a correspondence map between the samples of u and those of v.

The output of the algorithm is a series of pairs

(1) $$P\left(u,v\right)=\left((i(1),j(1)),\cdots ,(i(w),j(w))\right)\in {\left(N\times N\right)}^{w},w\in N,$$

which explicitly maps the indexes of each element of u with those of v. This path P(u,v), of length *w* is defined such that,

(2) $$\left(i\left(k\right),j\left(k\right)\right)\in P\left(u,v\right)\Leftrightarrow u_{i(k)}\;\mathrm{is}\;\mathrm{matched}\;\mathrm{with}\;v_{j(k)}.$$

**Figure 2:**
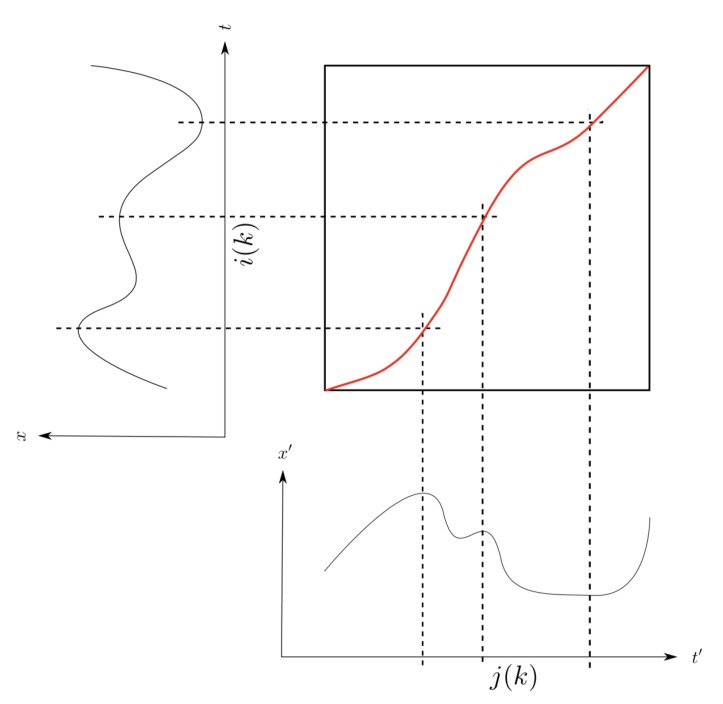
Two time series alignment: on the left the original signal u, at the bottom the signal v to be rematched, in the middle the realignment path *P* of between the two signals.

To illustrate this, we can represent the time series in a plane, with v on the horizontal axis, u on the vertical axis, and the path P(u,v) as a sequence of points in this two-dimension space (see Figure 2).

The accuracy of the correspondences induced by P(u,v) is intuitively assessed by the cost function W(P(u,v)) defined as:

(3) $$W\left(P\left(u,v\right)\right)=\sum_{k=1}^{w}d\left(u_{i(k)},v_{j(k)}\right),$$

where *d* is a distance over Ω. Note that W(P(u,v)) is non-negative and decreases with the similarity of the time series. In particular, if u and v are equal, $N_{u}=N_{v}$, the optimal path is $\left(1,1\right),\ldots ,\left(N_{u},N_{v}\right)$, and W(P(u,v))=0. The best alignment between two time series corresponds to the optimal path $P^{*}\left(u,v\right)$ that minimises Equation (3) over a finite set of paths P:

(4) $$P^{*}\left(u,v\right)=\underset{P(u,v)\in P}{\mathrm{argmin}}W\left(P\left(u,v\right)\right).$$

The aim of the DTW algorithm is to output the optimal path $P^{*}\left(u,v\right)$ given the two time series u and v and the distance *d*. The value of the cost function in Equation (3) obtained for this optimal path constitutes a measure of similarity, called the *DTW distance*

(5) $$\mathrm{DTW}\left(u,v\right)=W(P^{*}\left(u,v\right))$$

#### 2.2.2. Variants and Improvements

Over the years, many variants of the basic DTW algorithm have been introduced, mainly to increase its speed and lower its complexity [65,66,67,68]. We can for instance mention the FastDTW [66] or the SparseDTW [69]. Online algorithms have also been introduced in order to deal with time series whose size is not necessarily a priori known (such as, in a live recording [70]).

Importantly, some implementations of the DTW algorithm present an extra-parameter, maxsamp∈N, corresponding to the maximum difference acceptable between each match of each path. That is, for a any path P(u,v), we have:

(6) ∀(i(k),j(k))∈P(u,v),|j(k)−i(k)|<maxsamp.

The maxsamp parameter is here to ensure that the path *P* runs close to the diagonal of the distance matrix, as presented in Figure 2. It ensures that the warping does not align together too distant features, and limits the amount of distortion allowed in the re-alignment. It mainly allows a reduction of the DTW algorithm computation time, which is now quadratic in relation to maxsamp.

The application of the DTW algorithm thus becomes interesting in the search for a generalisable gait pattern in healthy subjects, through the signals of inertial sensors.

## 3. Data, Protocol and Subjects

### 3.1. Protocol

**Figure 3:**
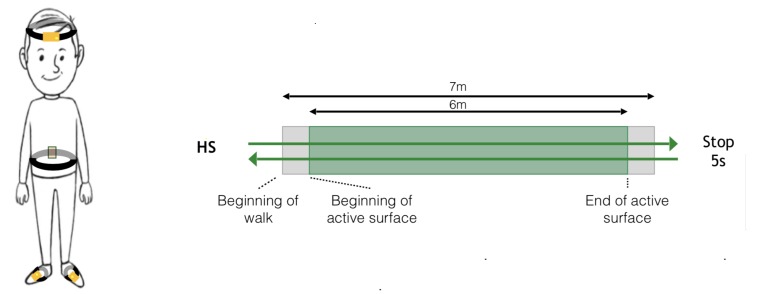
Gait Measure: synchronised Xsens-GaitRite. For healthy subjects, the U-turn is not on the mat.

Two XSens sensors (Xsens Technologies, Enschede, The Netherlands) (XS) were placed on the participant’s body (one on the dorsal part of each foot) using Velcro bands. The GaitRite walkway (GR) was used as the gold-standard. The data were sampled at 100 Hz for the XS and at 120 Hz for the GR. Both systems were synchronised in time by using the PC clock connected to XS. All subjects performed two recording sessions, one week apart. A last session was recorded, 3–6 months later, for a few subjects. Each session is composed of 42 direct passages on the GR. No U-Turn was considered in the recording. For practical reasons, subjects kept their own shoes. The protocol is schematised in Figure 3.

**Figure 4:**
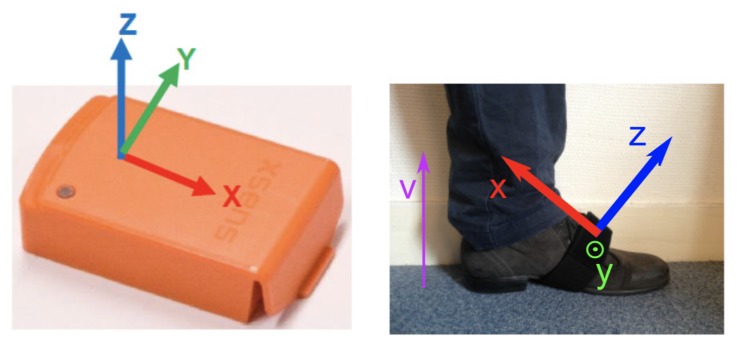
XSens Sensor, with axes orientations.

The protocol includes two sensors (left and right foot), and each of them records a nine-dimensional signal (3D accelerations, 3D angular velocities and 3D magnetic fields), possibly with some re-calibrated data provided by the XSens software (such as the vertical acceleration in the direction of the gravity), as presented in Figure 4. Instead of considering all these dimensions, we decided to only use one of them: the most relevant in the context of step detection. This decision was made based on observations of real data and physiological reasons provided by medical doctors. Consequently, in the following study, the y-axis angular velocity was the only component studied.

**Figure 5:**
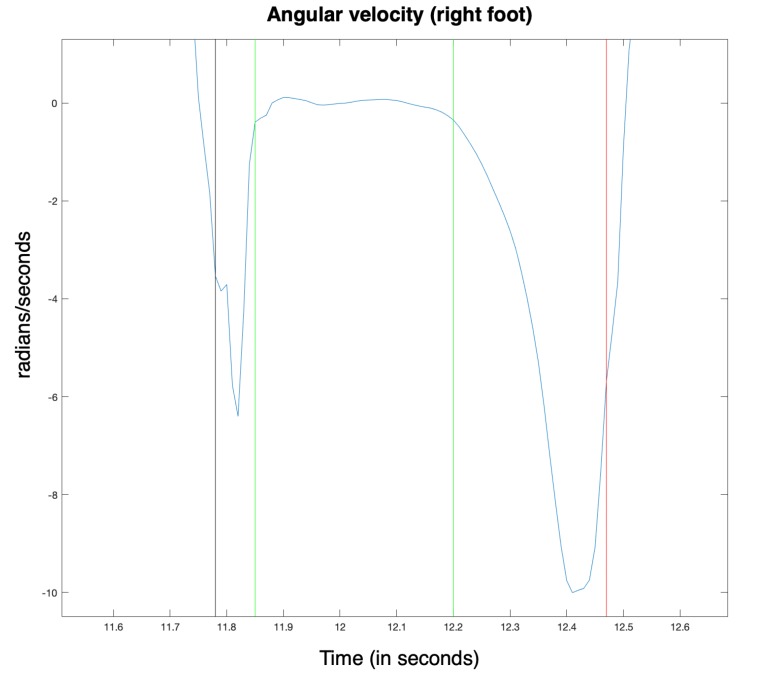
Key phases of the Gait Cycle, annotated on an y-axis angular velocity graphic. The events are represented by vertical lines. From left to right: “Heel Strike” (black), “Foot Flat” (green), “Heel Off” (green) and “Toe Off” (red).

Figure 5 represents a typical y-axis angular velocity signal, corresponding to a subject step, with annotated key instants.

### 3.2. Subjects and Database

The database is composed of 13 young healthy subjects who declared no medical impairment. All participants provided written informed consent before inclusion. The study protocol followed the principles of the Declaration of Helsinki. It was approved by the ethics committee “Protection des Personnes Nord Ouest III” (ID RCB: 2017-A01538-45). The main averaged characteristics of the subjects can be found in Table 1.

**Figure 6:**
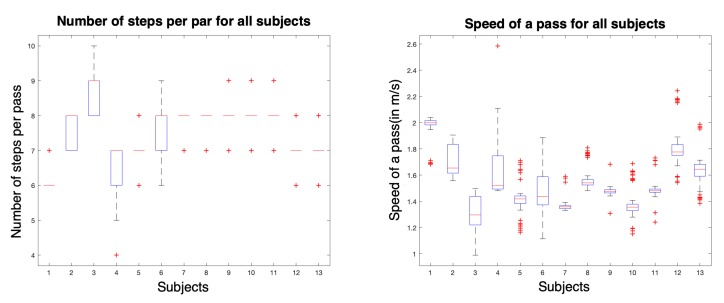
Box plots of the number of steps and the walking speed per subject.

Each subject performed the protocol approximately 20 times, which totals 7414 recorded steps. The average number of steps per trial is 7.5 steps, and the average speed is 1.5 m/s. The repartition of the number of steps and the average speed for each subject are displayed in Figure 6.

## 4. Method

In this section, we describe the two main contributions of this article:

- A new step detection algorithm based on template-matching and on a DTW refinement step
- Several strategies to construct the templates used in the algorithm

### 4.1. Dtw Step Detection

Our detection method is divided in two steps: a template-based step detection and a DTW refinement step.

First, a greedy template-based step detection algorithm is run, as described in [16]. This algorithm uses a library of templates to detect the steps. It computes a Pearson coefficient between each template and each position of the angular velocity signal, and then detects a step—after a threshold filtering, and if the template can be placed—around the Pearson coefficients local maxima. This algorithm outputs, for each step, the IC time $t_{IC}$ and the FC time $t_{FC}$. Each detected step is associated with one template p, which is the template in the library that was used to the detect this particular step. More specifically, given the input angular velocity signal x, the detected step $x_{t_{IC}:t_{FC}}$ is matched with template p and both have the same duration $t_{FC}-t_{IC}+1$.

Intuitively, if the library does not contain all possible step durations, this detection is inherently suboptimal. On the top of this first algorithm, we therefore propose in this article to add a refinement step, which searches for minimum DTW distances in the neighborhood of the previously detected steps, in order to increase the accuracy of the IC and FC detection of each step.

**Figure 7:**
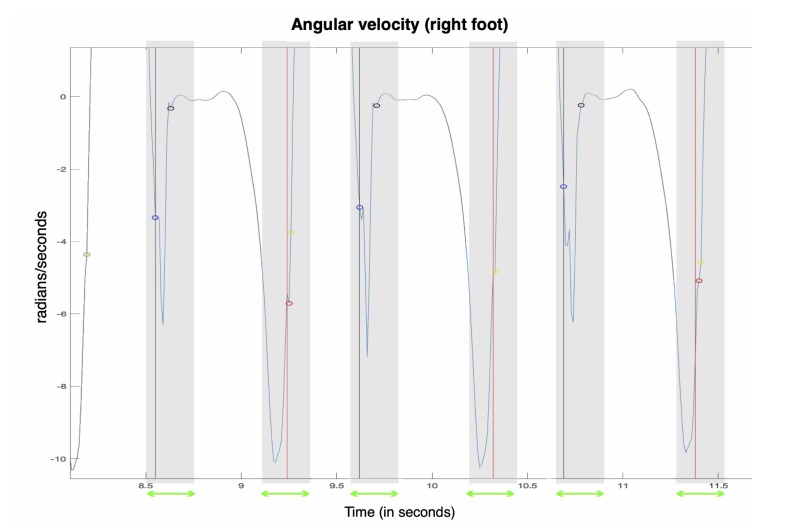
Around each step detected by template-matching, a 2×z samples-sized area (green arrows, grey zones) is scanned (in this case, z=10) and DTW distances are computed for each one of these samples. The black and red points correspond to the IC and FC instants predicted by template matching. The blue and yellow points correspond to the IC and FC instants detected after the DTW refinement step. The black and red lines correspond, respectively, to the IC and FC of the step, as annotated by the GR walkway.

The DTW refinement step is the following one. Once a step is detected by template-matching, we scan the area near the detection in order to find the smallest DTW distance between the match and the template: i.e., to find the signal the most similar to the template, structurally. Technically, we scan a +/−*z* samples area around the detected IC and FC, to take into account all the different lengths of the steps, compared to the template length. Thus, ${\left(2z+1\right)}^{2}$ combinations are tested for each detected step. Figure 7 presents an illustration of the process.

Given a lag $\left(k,l\right)\in {\left[\;\left[-z,z\right]\;\right]}^{2}$, we compute the DTW distance between the original template p and the shifted step $x_{t_{IC}+k:t_{FC}+l}$ and find the lag $\left(k^{*},l^{*}\right)$ that minimises this distance:

(7) $$\left(k^{*},l^{*}\right)=\underset{\left(k,l\right)\in {\left[\;\left[-z,z\right]\;\right]}^{2}}{\mathrm{argmin}}\;\mathrm{DTW}\left(x_{t_{IC}+k:t_{FC}+l},p\right)$$

The new IC/FC times after refinement are defined as $t_{IC}^{*}=t_{IC}+k^{*}$ and $t_{FC}^{*}=t_{FC}+k^{*}$.

### 4.2. Construction of the Library of Templates

In this article, we investigate five different strategies for constructing the library of templates. For all strategies, the library of template is constructed from the library of 7414 steps annotated by the GaitRite.

#### 4.2.1. Strategy 1: Random Selection

The first strategy consists in randomly choosing a certain number of steps from the train library and using them to detect the steps in the other signals. We propose to compare two library sizes: for S1-10, ten templates are chosen and, for S1-1, only one template is used.

#### 4.2.2. Strategy 2: DTW-Based Selection

An alternative strategy consists in choosing one specific template from the train library, by selecting the more appropriate according to the step detection task. A first approach in this search for an ideal template would be to take the step, in the whole database, which is, structurally, the nearest to all the other steps. If the DTW distance is considered as an effective measure of similarity between time series, this step would correspond to the step with the minimum mean-DTW-distance to all the other steps of the database.

Considering the train library $S_{train}$ composed of $N_{train}$ steps $s^{\left(1\right)},\ldots ,s^{\left(N_{train}\right)}$, we choose the step $s^{*}$ such that

(8) $$s^{*}=\underset{s\in S_{train}}{\mathrm{argmin}}\sum_{n=1}^{N_{train}}\mathrm{DTW}\left(s,s^{\left(n\right)}\right)$$

**Figure 8:**
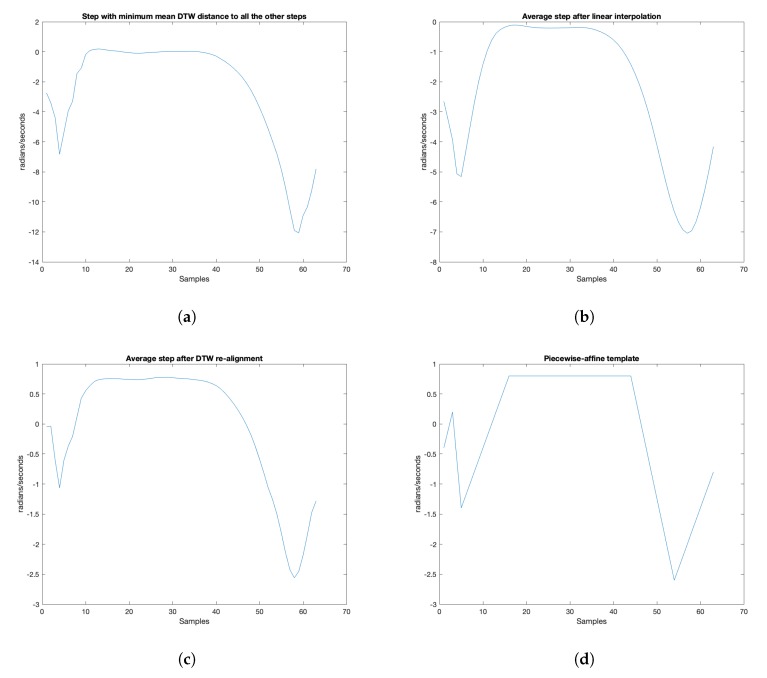
Learned templates for strategies S2, S3, S4 and S5. All templates contain 63 samples, which is the median length of the steps in the database. (**a**) Strategy S2: DTW-based selection; (**b**) Strategy S3: Linear fusion; (**c**) Strategy S4: Non-linear fusion; (**d**) Strategy S5: Knowledge-based approximation.

In strategy S2, the unique used template $s^{*}$ is the nearest, by a DTW point of view, to all the steps from the database. The result for strategy S2 applied on our database can be seen in Figure 8a.

#### 4.2.3. Strategy 3: Linear Fusion

Another approach would be to merge all steps from the train database to compute one unique template that reflects the most shared properties. This process was already used by Soaz and Diepold [17] to obtain the characteristic steps of some particular gait clusters. Merging all steps necessitates first applying a time and amplitude normalisation, which can be performed either linearly or non-linearly. In strategy S3, we take all steps from the database, normalise them so that they have zero mean and unit variance, resample them linearly so that their durations are equal to the median duration of 63 samples and average them.

The result for strategy S3 applied on our database can be seen in Figure 8b.

#### 4.2.4. Strategy 4: Non-Linear Fusion

Another method to obtain an average step is to re-align all the steps, by DTW, on a single one, and then to average them all. Contrary to strategy S3, the time normalisation in this context is non-linear since the DTW resamples the signals through a non-uniform sampling.

Before anything, all the steps of the database are normalised in amplitude so as to have zero mean and unit variance, as in S3. Then, we select all the steps of length equal to the median length of our steps database (63 samples), and then select the one with the minimum mean DTW distance to all the database steps (in a process analogous to the one exposed in strategy S2). The chosen step $s^{c}$, referred to as the *calibration step*, serves as a mold on which all steps will be blended.

The non-linear time renormalisation based on DTW performs as follows. Given a step s of duration $N_{s}$ and the calibration step $s^{c}$ of duration $N_{c}=63$, the renormalised step $s^{\prime }$ is computed such that for $i\in [\;\left[1,N_{c}\right]\;]$

(9) $$s_{i}^{\prime }=\frac{1}{\mathrm{Card}\{j|\left(i,j\right)\in P^{*}\left(s^{c},s\right)\}}\sum_{\left\{j|\left(i,j\right)\in P^{*}\left(s^{c},s\right)\right\}}s_{j},$$

where $P^{*}\left(s^{c},s\right)$ is the optimal path between $s^{c}$ and s (see Equation (4)). In other words, each sample $s_{i}^{\prime }$ corresponds to the mean of all the samples $s_{j}$ matched with $s_{i}^{c}$ on $P^{*}\left(s^{c},s\right)$.

The result for strategy S4, applied on our database, can be seen in Figure 8c.

#### 4.2.5. Strategy 5: Knowledge-Based Piecewise-Affine Approximation

The last strategy consists in building the template by hand by using biomechanical assumptions of the shape of the angular velocity during a step. To that end, we used assumptions described in Section 2.1 on gait events. Based on biomechanical considerations, we placed all gait events (HS, TO, etc.) with amplitudes similar to the ones observed in strategies S2, S3 and S4 and assumed a simple affine model between them. The so-formed template can be seen as an abstraction or idealised step. Similar to strategies S3 and S4, its length is equal to the median length of the steps database, i.e., 63 samples.

Its equation is as follows:

(10) $$f\left(x\right)=\left\{\begin{array}{cc} \; & 0.3x-0.7\;\mathrm{if}\;x\in [1;3]\\ \; & -0.8x+2.6\;\mathrm{if}\;x\in [3;5]\\ \; & 0.2x-2.4\;\mathrm{if}\;x\in [5;16]\\ \; & 0.8\;\mathrm{if}\;x\in [16;44]\\ \; & -0.34x+15.76\;\mathrm{if}\;x\in [44;54]\\ \; & 0.2x-13.4\;\mathrm{if}\;x\in [54;63] \end{array}\right.$$

This piecewise-affine step is represented in Figure 8d.

### 4.3. Evaluation Metrics

The following metrics are used to evaluate our process and the different strategies. They use as ground truth the annotations given by the GR walkway. For the evaluation, a step is defined as the period between IC and FC (which corresponds to the stance phase); the start time refers to the IC and the end time to the FC.

- Precision. A detected step is counted as correct if the mean of its start and end times lies inside an annotated step. An annotated step can only be detected one time. If several detected steps correspond to the same annotated step, all but one are considered as false. The precision is the number of correctly detected steps divided by the total number of detected steps.
- Recall (or sensitivity). An annotated step is counted as detected if the mean of its start and end times lies inside a detected step. A detected step can only be used to detect one annotated step. If several annotated steps are detected with the same detected step, all but one are considered undetected. The recall is the number of detected annotated steps divided by the total number of annotated steps.
- ΔStart. For a correctly detected step, it is the difference between the detected start time and the annotated start time.
- ΔEnd. For a correctly detected step, it is the difference between the detected end time and the annotated end time.
- ΔDuration. For a correctly detected step, it is the difference between the duration of the detected step and the duration of the annotated step.

### 4.4. Experiments

The DTW algorithm used in our process is the one implemented in Matlab Version R2019a.

The width of the warping window maxsamp was set to 20 samples. The length of the DTW scan area *z* in Equation (7) presented in Section 4.1 was set to z=10. The influence of parameter *z* is discussed in Section 6.3. The pointwise distance d(.,.) used in Equation (3) was chosen as

(11) $$d\left(u_{i},v_{j}\right)={\left(\frac{u_{i}-\bar{u}}{\sigma_{u}}-\frac{v_{j}-\bar{v}}{\sigma_{v}}\right)}^{2},$$

where $\bar{.}$ and $\sigma_{.}$ are, respectively, the mean and standard deviations of time series u and v. This distance ensures that the input signals are first renormalised with zero mean and unit variance.

For the random strategies S1-1 and S1-10, 20 independent simulations were computed and signals that were used for training were removed from the test database. In both cases, the evaluation metrics of the 20 simulations were concatenated and means and standard deviations are displayed.

To investigate the influence of the DTW refinement step and of the different strategies for choosing the templates, we ran the following experiments.

- **Experiment 1.** For the S1-1 and S1-10 strategies, we studied the influence of the DTW refinement step described in Section 4.1 by comparing the metrics obtained with and without this additional step.
- **Experiment 2.** We compared the metrics obtained by strategies S2, S3, S4 and S5 by using the step detection algorithm with the DTW refinement step.
- **Experiment 3.** For strategy S5, we studied the influence of parameter *z* by computing the evaluation metrics ΔStart, ΔEnd and ΔDuration for various values of *z* from 1 to 20 samples.
- **Experiment 4.** For strategy S5, we compared the evaluation metrics ΔStart, ΔEnd and ΔDuration, obtained after a DTW refinement step, and a linear-correlation refinement step. To implement the linear-correlation refinement step, we re-implemented the process described in Section 4.1, replacing the search for a minimum DTW distance by the search for a maximum Pearson coefficient.

## 5. Results

### 5.1. Comparison with State-Of-The-Art

Gait event detection is a hot topic that has been widely studied in the literature. We refer the readers to [4,6,71,72] for a comprehensive survey of the state-of-the-art methods dedicated to this task. A recent review article published in 2019 displays a comparison of all main approaches for IC/FC detection from a single IMU on healthy adults subjects [73]. Reported scores for ΔStart range from 0.012 to 0.072 s depending on the method and from 0.012 to 0.112 s for ΔEnd. These values are in accordance with several recent publications on the topic (≥ 2019): Caramia et al. [74] (ΔStart = 0.022 s, ΔEnd = 0.024 s), Kidzinski et al. [35] (ΔStart = 0.010 s, ΔEnd = 0.013 s), Gadaleta et al. [34] (ΔStart ≈ΔEnd ≈ 0.040 s) and Mei et al. [75] (ΔStart ≈ΔEnd ≈ 0.020 s). These values (summarised in Table 2) are to be compared with the results presented in this section.

### 5.2. Experiment 1

A sum-up of the evaluation metrics obtained for the S1-1 and S1-10 strategies, after 20 independent simulations, with or without the DTW refinement step, can be seen in Table 3. For the S1-1 strategy, the DTW refinement step divides by approximately two the ΔStart and ΔDuration evaluation metrics. For the S1-10 strategy, the impact is limited. Computation times for processing the whole database (7414 steps) is displayed in Table 3.

### 5.3. Experiment 2

A sum-up of the evaluation metrics obtained by strategies S2, S3, S4 and S5 presented in Section 4.2, by using the detection algorithm with the DTW refinement step, are presented in Table 3. All five strategies give more accurate evaluation metrics than the S1-10 strategy, after the DTW refinement step, as presented in Section 5.2.

### 5.4. Experiment 3

**Figure 9:**
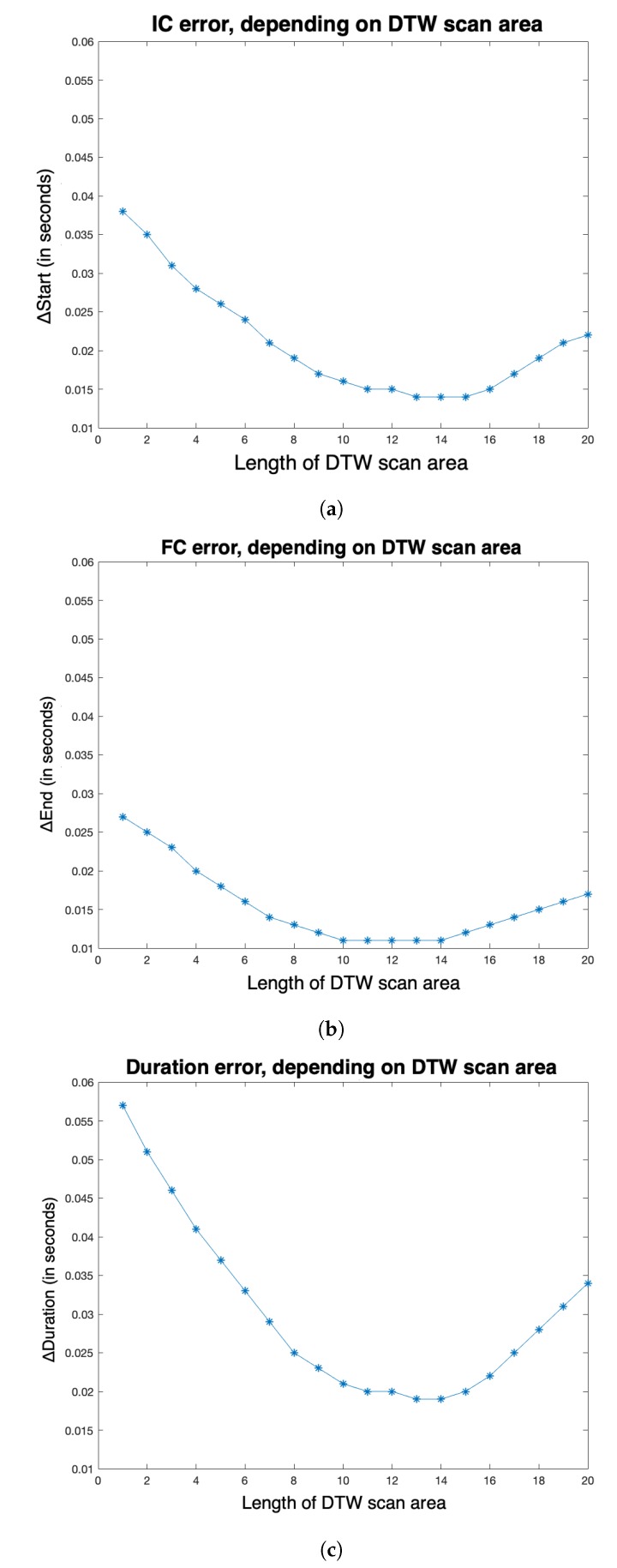
For strategy S5, influence of parameter z on the evaluation metrics (**a**) ΔStart, (**b**) ΔEnd and (**c**) ΔDuration, for z varying from 1 to 20.

For strategy S5, the influence of parameter *z* on the evaluation metrics ΔStart, ΔEnd and ΔDuration was studied, for *z* varying from 1 to 20, in Figure 9a–c. A minimum value of ΔStart, ΔEnd and ΔDuration is reached for z=13.

### 5.5. Experiment 4

**Figure 10:**
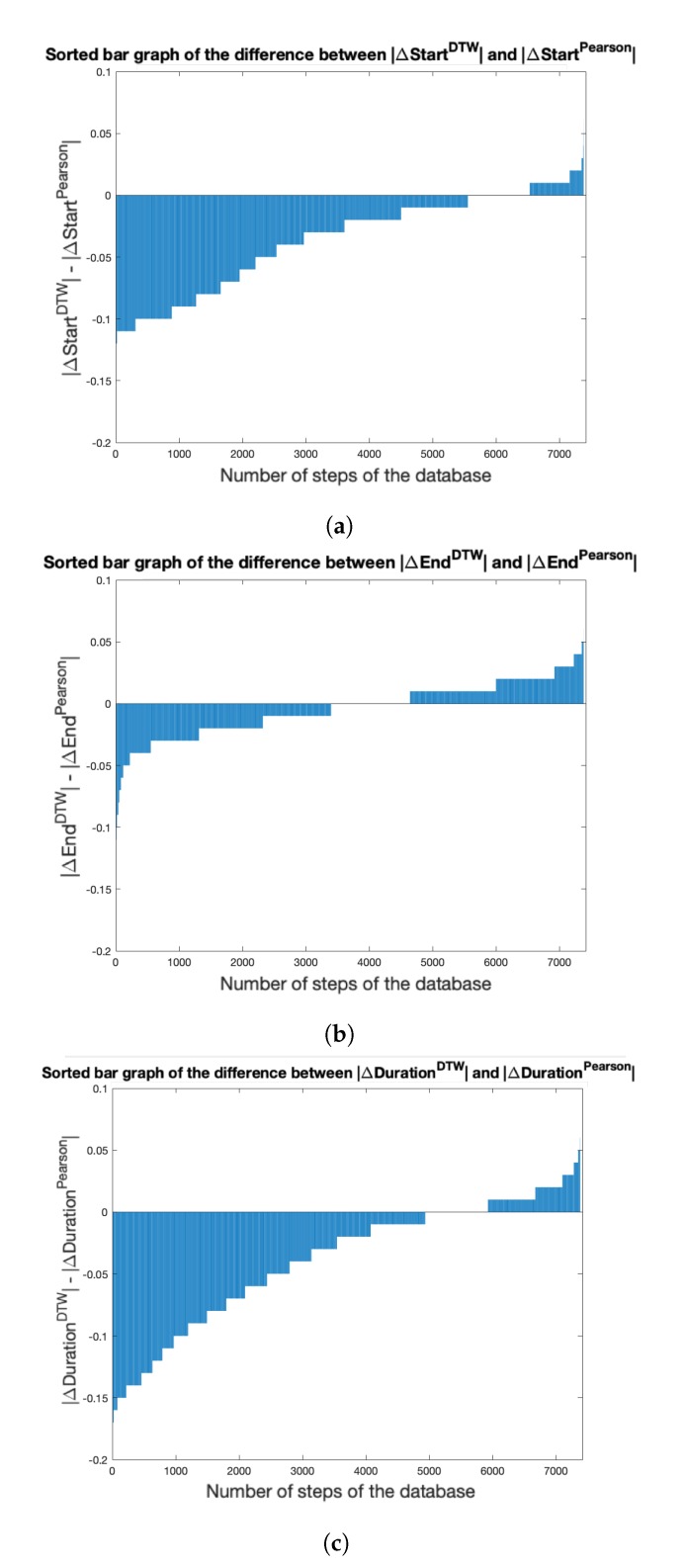
For strategy S5, sorted bar graph of the difference between the 7414 (**a**) ΔStart, (**b**) ΔStart and (**c**) ΔDuration absolute errors obtained with the DTW distance criterion, and those obtained with the Pearson coefficient criterion.

For strategy S5, we compared the evaluation metrics ΔStart, ΔEnd and ΔDuration, obtained after a DTW refinement step, and a linear-correlation refinement step. The results, presented as the difference of the absolute evaluation metrics ΔStart, ΔEnd and ΔDuration obtained by each method, can be seen in Figure 10a–c. The DTW refinement step gives better ΔStart, ΔEnd and ΔDuration than the linear-correlation refinement step.

## 6. Discussion

### 6.1. Experiment 1

If the effect of the DTW refinement step is not striking in the S1-10 strategy, it is particularly strong in the S1-1 strategy. Indeed, S1-1, with the DTW refinement step, gives a result almost comparable with S1-10, with or without the DTW refinement step.

A single template step, correctly re-aligned, is able to accurately detect all the steps of our HS database. This means that all the HS of our database share a common gait pattern, but, also, that the step deformations between each subject follows a non-linear deformation comparable to a DTW deformation. Previously, the authors have highlighted the impact of immobilisation of a joint on the other joints of the lower limb, depending on the phases of the step [76]. Thus, the joints were not affected in the same way at the different phases of gait, which tends to underline the non-linearity of the gait pattern. This non-linearity questions the methods of detecting steps using threshold values [77] since these values may not be proportional in duration or amplitude with a reference value necessary for establishing the threshold. Maintaining a key gait parameter, such as gait velocity, could lead to global and non-linear changes in other joints, resulting in internal dynamics of adaptation. The emergence of these stereotypical adaptation patterns could result from, for example, the search for control of an appropriate parameter through physical rehabilitation [78,79]. The existence of a scheme as simple as the one we have drawn, whose non-linear modifications make it possible to maintain a functional gait, is in line with the theories of Winter and those who have succeeded him, i.e., that the gait must ensure a minimum of invariant subtasks whose intermediate stages are subjected to deformations or modulations depending on the context [79]. Our algorithm would find these invariants here and adapt the signal to the complex and dynamic modulations offered by the peripheral nervous system and the supraspinal inputs [80]. The use of generative walking patterns in the field of rehabilitation has already been the subject of publications, highlighting the benefits of a layer-pattern with flexible and adjustable parameters [81].

### 6.2. Experiment 2

The four strategies S2, S3, S4 and S5 present, after the DTW refinement step, comparable, very accurate evaluation metrics. Importantly, they all give more accurate metrics than the average S1-10 strategy, with or without DTW, tested in Section 5.2. One striking fact is that strategy S5, based on a piecewise-affine step template, inferred from biomechanical knowledge, as presented in Section 4.2.5, presents highly accurate metrics (in fact, it is the tested strategy giving the best evaluation metrics, on average). This result is rich in conclusions. Indeed, if all the steps of our HS database can be very accurately detected with such a simple template, it means that human locomotion is a phenomenon extremely similar, in its nature, between every healthy subject. Even if steps of healthy subjects highly change in duration, etc., they can be approximated by a very schematic template, which, with a correct method, is able to fit them all. Moreover, it means that the biomechanical assumptions on which we based the creation of this template were a priori adapted, and shared by all the subjects of our database.

### 6.3. Experiment 3

As we could expect, for S5, ΔStart, ΔEnd and ΔDuration decrease and then increase with the length of *z*, the DTW scan area. The three curves present a minimum for a DTW scan area length *z* equal to 13 samples. At the sight of these curves, the 10-sample length choice, for *z*, presented in Section 4.4 appears acceptable: for S5, the three evaluation metrics are low for this value of DTW area length. Even if a choice of *z* length equal to 13 samples would have certainly given more accurate results, it would have significantly increased the computation times of our simulations.

### 6.4. Experiment 4

For S5, the results are significantly better with the DTW distance refinement step than with the linear correlation refinement step. This is particularly striking for the evaluation metrics ΔStart and ΔDuration; indeed, it appears that the template-matching algorithm presented in Section 4.1 naturally fits templates at the end of the detected steps, and not at the beginning. This result could have been expected: indeed, the length of each gait phase heavily depends on the subject’s characteristics (age, medical conditions, etc.), as well as on contextual characteristics, such as the speed of the walk. Even if the gait phases are represented by similar patterns on the y-axis angular velocity graphics, their durations therefore essentially vary. To take into account those non-linear variations, the DTW distance criterion seems logically way better adapter than a linear correlation criterion. These results are also in accordance with Experiment 1: the appropriate tool for modeling the typical deformations of any universal gait template should include the possibility for non-linearities.

### 6.5. Computation Time

As presented in Section 2.2.1, our DTW algorithm computation time is quadratic in relation to *maxsamp*. On our own setup (2 GHz Intel Core i5 processor) and a non-optimised implementation of DTW, the computation times for all strategies can be found in Table 3. For strategy S1-1, the DTW refinement step approximately increases computation time by a factor 4. Strategies S2, S3, S4 and S5 have similar computation times as strategy S1-1. For strategy S1-10, the DTW refinement step approximately increases computation time by a factor 7. Even if the DTW refinement step is computationally expensive, its computation times are not excessive since, as shown in Section 6.1, the use of DTW allows to only use one template instead of ten, with better performances. This computational time can also been reduced by using recent approximate variants of DTW [54].

### 6.6. Limitations

The main limitations of this study are linked to the number of subjects and to the relative homogeneity of the considered database (healthy subjects). Further studies on elderly or pathological subjects will be of high interest to investigate if the conclusions of the present study are also valid for different populations or on a larger range of subjects. Besides, other detection methods could have been tested. For instance, Discrete Wavelet Transform (DWT)/Continuous Wavelet Transform (CWT) use for gait/ECG phases detection has been advocated in numerous articles [82,83,84]. It is nevertheless to be supposed that these methods, based on linear scale deformations—such as the linear correlation criterion—would not have given as good results as the DTW distance criterion.

## 7. Conclusions

Thanks to the method presented in this article, based on a greedy template-matching algorithm and a DTW refinement step, a single step template is enough to accurately detect all the steps of a healthy subjects database. However, this detection is here possible due to two main contributions: the use of a non-linear measure of fit (DTW) that allows comparing time series of different lengths and the use of a relevant template inferred from real physiological data. We believe that these results will be of interest for the neurological interpretation of locomotion and further studies shall be conducted to confirm/infirm these conclusions on other populations.

## Figures and Tables

**Table 1:** Baseline characteristics of studied Healthy Subjects. For age and height, the mean and standard deviations are displayed.

	Subjects (n = 13)
Sex (M/F)	6/8
Age (years)	26.6 (2.0)
Height (m)	1.69 (0.09)
Weight( kg)	63.3 (14.8)

**Table 2:** State-of-the-art results: ΔStart and ΔEnd are displayed in milliseconds. Note that these results are only displayed for information since the used metrics, sensors and databases are not the same according to the publication. They only provide an order of magnitude of current performances in the literature.

Publication	ΔStart	ΔEnd
Perez et al. (2019) [73]	12 to 72	12 to 112
Caramia et al. (2019) [74]	22	24
Kidzinski et al. (2019) [35]	10	13
Gadaleta et al. (2019) [34]	≈ 40	≈ 40
Mei et al. (2019) [75]	≈ 20	≈ 20

**Table 3:** Evaluation metrics obtained by strategies S1, S2, S3, S4 and S5. For S1-1 and S1-10, the precision and recall are averaged on the whole database and on 20 independent simulations (means and standard deviations). ΔStart, ΔEnd and ΔDuration are displayed in milliseconds and averaged on the whole database (means and standard deviations). Computation time corresponds to the time to process the whole database.

		Precision	Recall	ΔStart	ΔEnd	ΔDuration	Computation Time
**S1-1**	no DTW	0.99 (0.012)	0.96 (0.086)	49 (63)	15 (21)	60 (74)	8 min
	DTW	0.99 (0.012)	0.96 (0.086)	25 (30)	13 (17)	27 (33)	33 min
**S1-10**	no DTW	0.99 (0.008)	1.0 (0.0)	16 (23)	13 (18)	23 (32)	37 min
	DTW	0.99 (0.008)	1.0 (0.0)	20 (25)	12 (17)	24 (29)	4 h 20 min
**S2**	DTW	1.0	1.0	21 (23)	11 (14)	26 (25)	33 min
**S3**	DTW	0.96	0.97	17 (25)	15 (14)	18 (26)	33 min
**S4**	DTW	1.0	1.0	21 (23)	10 (13)	22 (25)	33 min
**S5**	DTW	0.99	0.99	15 (18)	16 (13)	19 (15)	33 min
